# Methylprednisolone-induced anaphylaxis diagnosed by intradermal skin test: a case report

**DOI:** 10.1186/s13223-021-00570-1

**Published:** 2021-07-13

**Authors:** Hitomi Amano, Yoshiro Kitagawa, Tomohito Hayakawa, Taichiro Muto, Akihisa Okumura, Hideyuki Iwayama

**Affiliations:** 1 Postgraduate Clinical Training Center, Aichi Medical University Hospital, Nagakute, Aichi Japan; 2 Department of Pediatrics, School of Medicine, Aichi Medical University, 1-1, Yazakokarimata, Nagakute, Aichi 480-1195 Japan; 3 Nagakute Kitagawa Children’s Clinic, Nagakute, Aichi Japan

**Keywords:** Methylprednisolone-induced anaphylaxis, Glucocorticoids, Immediate allergy, Succinate ester, Basophil activation test, Intradermal skin test

## Abstract

**Background:**

Glucocorticoids rarely cause anaphylaxis. Common methods for the determination of allergens include in vivo skin prick test (SPT) and intradermal skin test (IDST) and the in vitro basophil activation test (BAT). However, to our knowledge, the best strategy for diagnosing glucocorticoid-induced anaphylaxis has not been elucidated.

**Case presentation:**

A 10-year-old boy was admitted to our hospital because of 2 weeks of fever and arthralgia. He had not been treated with glucocorticoids before, including methylprednisolone (mPSL). He was suspected to have bacterial myositis and was treated with ceftriaxone. However, his symptoms persisted for > 2 weeks. Autoinflammatory arthritis was suspected, and he was treated with mPSL sodium succinate (MPS) pulse therapy (30 mg/kg). After 15 min of mPSL injection, he had wheezing and generalized wheal formation with decreased oxygen saturation. As anaphylaxis was suspected, mPSL was discontinued, and olopatadine and oxygen were administered. The symptoms improved considerably without the use of epinephrine and disappeared in 30 min. One month after discharge, SPT, IDST, and BAT were performed without discontinuing his prescribed oral prednisolone. SPTs for MPS, hydrocortisone sodium succinate (HCS), prednisolone sodium succinate (PSS), dexamethasone sodium phosphate (DSP), and betamethasone sodium phosphate (BSP) were negative. IDSTs for MPS, HCS, and PSS were positive, whereas those for DSP and BSP were negative. By contrast, BATs for MPS, HCS, and PSS were negative. Although glucocorticoid-induced hypersensitivity caused by nonmedicinal ingredients such as lactose, carboxymethylcellulose, polyethylene glycol, and hexylene glycol has been reported; the glucocorticoids tested in this patient did not contain any of these nonmedicinal ingredients. As the glucocorticoids that were positive on IDST share a succinate ester, this might have caused MPS-induced anaphylaxis.

**Conclusions:**

We report the case of MPS-induced anaphylaxis diagnosed by IDST but not BAT. In case reports of glucocorticoid-induced anaphylaxis in the literature, most patients were diagnosed with SPT or IDST. These results suggest that BAT should be considered when IDST and SPT are negative. Further studies are necessary to clarify the best strategy for diagnosing glucocorticoid-induced anaphylaxis.

## Background

Glucocorticoids are usually used to treat anaphylaxis. However, glucocorticoids rarely cause anaphylaxis. Glucocorticoids are hydrophobic particles, and their intravenous forms have succinate ester that make them hydrophilic. Anaphylaxis secondary to glucocorticoids that contain succinate ester are reported to have a prevalence of about 0.3%, which is not unexpectedly rare [1].

Determination of the cause of allergy is critical for proper care. This analysis includes in vivo tests, such as the skin prick test (SPT) and intradermal skin test (IDST), and in vitro tests, such as the allergen-specific IgE test and basophil activation test (BAT). Allergen-specific immunoglobulin E (IgE) test is not a reliable test for glucocorticoid-induced allergy, as mentioned earlier [2]. BAT is a flow cytometry-based assay that measures the expression of activation markers on the surface of basophils following stimulation with an allergen [3]. The usefulness of BAT has been studied well for the determination of food allergens [3]. However, BAT has been reported in only a limited number of cases of glucocorticoid-induced anaphylaxis [4–8]. Therefore, the diagnostic strategy for glucocorticoid-induced anaphylaxis has been elucidated.

In this study, we reported the case of a boy who developed anaphylaxis secondary to intravenous methylprednisolone (mPSL) sodium succinate (MPS) during pulse therapy. The drug allergy was proven by IDST, not BAT.

## Case presentation

A 10-year-old boy was admitted to our hospital because of 2 weeks of fever and arthralgia. His medical history was unremarkable, and he had not been previously treated with glucocorticoids, including mPSL. Laboratory testing showed leukocytosis of 22,400/mL with 88% segmented neutrophils and an elevated C-reactive protein of 9.21 mg/dL. Magnetic resonance imaging of the lower extremities with fat-suppressed T2-weighted images showed high intensity in the right gluteus maximus muscle. He was suspected to have bacterial myositis and was treated with ceftriaxone. However, his symptoms persisted for more than 2 weeks. Autoinflammatory arthritis was suspected, and treatment with mPSL pulse therapy (30 mg/kg) was initiated.

After 15 min of mPSL injection, he had wheezing and generalized wheal formation with decreased oxygen saturation. As anaphylaxis was suspected, mPSL was discontinued, and olopatadine and oxygen were administered. The symptoms improved considerably without the use of epinephrine and disappeared in 30 min. Oral prednisolone (PSL, 2 mg/kg/day) was started for the treatment of arthritis and afforded resolution of fever and arthralgia. He was discharged from the hospital 1 week after the start of oral PSL. There was no relapse and oral PSL was reduced to 5 mg/day after 1 year.

One month after discharge, SPT, IDST, and BAT were performed to assess for reaction to glucocorticoids including MPS. Of note, the patient remained on his prescribed oral PSL during this evaluation. SPTs for MPS, hydrocortisone sodium succinate (HCS), PSL sodium succinate (PSS), dexamethasone sodium phosphate (DSP), and betamethasone sodium phosphate (BSP) were negative. IDST for MPS resulted in a 7 × 7-mm wheal and a 9 × 8-mm area of erythema, whereas normal saline revealed no wheal or erythema. Therefore, IDST for MPS was positive. Moreover, IDSTs for HCS and PSS were also positive, whereas those for DSP and BSP were negative. BAT for MPS was negative compared to the positive control stimulated by a monoclonal anti-IgE antibody (1-h incubation, 1.0% vs 60.4%; 24-h incubation, 1.9% vs 18.3%). Nonmedical ingredients in the glucocorticoids tested in this patient are shown in Table 1.

**Table 1:** Nonmedicinal ingredients in the glucocorticoids used in skin tests

Glucocorticoid	Na2HPO4	NaH2PO4	NaOH	Others
Methylprednisolone sodium succinate	+	+	+	
Prednisolone sodium succinate	+	+	−	Na2CO3
Hydrocortisone sodium succinate	+	+	+	
Dexamethasone sodium phosphate	+	−	+	NaHSO3, NaCl, Sodium citrate
Betamethasone sodium phosphate	+	+	−	d-sorbitol, Na2SO3

## Discussion and conclusions

### Anaphylaxis to glucocorticoids

Nonmedicinal ingredients such as lactose, carboxymethylcellulose, polyethylene glycol, and hexylene glycol are reported causes of glucocorticoid-induced hypersensitivity [2]. The glucocorticoids tested in this patient did not contain any of these nonmedicinal ingredients (Table 1). Since the glucocorticoids that elicited positive IDSTs share a succinate ester, we diagnosed anaphylaxis secondary to the succinate ester in MPS.

Succinate ester was found to be the cause in most cases of anaphylaxis secondary to systemic glucocorticoid administration [9]. Our patient had anaphylaxis induced by MPS during mPSL pulse treatment for autoinflammatory arthritis. Succinate is the common chemical structure among MPS, HCS, and PSS. Notably, IDSTs for all three drugs were positive in this patient, indicating that the anaphylaxis was attributable to succinate.

As the patient had not previously been exposed to glucocorticoid medications, he must have been exposed and sensitized to succinate ester in another form before the episode of anaphylaxis. Some food additives contain succinate ester. For example, octenyl succinic acid-modified starch is listed in the General Standard for Food Additives for use as a stabilizer, emulsifier, and thickener in several food categories [10]. Therefore, it is possible that our patient consumed these food additives and was sensitized without noticing. No study reported the cross-reactivity between succinate and phosphate ester, indicating that cross-reactivity between the two esters may not be the cause of sensitization in this patient.

### SPT, IDST, and BAT

In vivo tests, such as SPT and IDST, are generally used for determining the causative drugs in patients with anaphylaxis [3], although BAT has been reported to be useful in proving anaphylaxis secondary to MPS [5]. We searched for case reports on the use of BAT for the diagnosis of glucocorticoid-induced anaphylaxis in PubMed and Ichushi-Web, which is the Japanese database of medical literature updated by the Japan Medical Abstracts Society (Table 2) [4–8]. Among the 10 patients, including ours, BAT was positive in six and negative in four. IDST was negative in one patient and SPT in five. In the four patients with a negative BAT, IDST was positive in two and SPT in three. These facts suggest that a combination of diagnostic tests is necessary to diagnose glucocorticoid-induced anaphylaxis.

**Table 2:** Case reports of BAT for steroid-induced anaphylaxis in the literature

No.	Authors	Year	Age	Sex	Primary disease	Steroids	BAT	SPT	IDST	Challenge test
1	Ben Said [5]	2007	18	F	General anesthesia	MPS	Positive	Positive	Positive	ND
2	Lehmann [8]	2008	2	M	Bronchial asthma	PSS	Positive	Negative	Positive	ND
3	Aranda [4]	2010	22	F	Dental infection	MPS	Positive	Positive	ND	ND
4			32	M	Parotitis	MPS	Negative	Positive	ND	ND
5			46	F	Dental infection	MPS	Positive	Negative	Negative	Positive
6			65	F	Dental surgery	MPS	Positive	Negative	Positive	ND
7	Walker [7]	2011	26	M	Bee bite	PSS, MPS	Negative	Positive	ND	Positive
8			70	M	Sudden deafness	PSS, HCS	Positive	Negative	Positive	ND
9	Nucera [6]	2011	45	F	Dialysis surgery	HCS	Negative	Positive	Positive	ND
10	Amano (this study)	2020	10	M	Autoinflammatory arthritis	MPS	Negative	Negative	Positive	ND

*M* male, *F* female, *MPS* methylprednisolone sodium succinate, *PSS* prednisolone sodium succinate, *HCS* hydrocortisone sodium succinate, *SPT* skin prick test, *IDST* intradermal skin test, ND not described

In our patient, BAT was negative for glucocorticoids that contained succinate ester regardless of incubation time, whereas IDST was positive. This may be explained by the use of an oral glucocorticoid during blood sampling for BAT. Individuals being tested on BAT should stop treatment with oral steroids 3 weeks before the test [11], although no significant reduction of basophil reactivity was observed at the concentration of the therapeutic range of prednisolone [12]. PSL may reduce basophil reactivity to succinate ester by different mechanisms in 1-h and 24-h incubations. The rapid inhibitory effect of glucocorticoids through a nongenomic pathway induces decreased basophil activity [13], which may be the cause of a negative BAT at 1 h. On the other hand, glucocorticoids are reported to induce DNA fragmentation and apoptosis of basophils for several hours through modulation of gene expression [14], which may be the cause of the negative BAT 24 h after incubation. Although BAT is known to be safe [3], its diagnostic value in patients with MPS-induced anaphylaxis should be elucidated, especially when the patient is taking glucocorticoids. Another possible explanation for the negative BAT in this patient is a non-IgE-mediated hypersensitivity to MPS. Hypersensitivity reactions to drugs are often type I, but they can be type II, III, or IV [15]. If the result of BAT is accurate, then the possibility of an IgE-mediated allergy is unlikely. However, considering the time between drug administration and the onset of anaphylaxis, type 1 allergy is the most likely cause of anaphylaxis in this case.

In conclusion, IDST was useful in establishing the diagnosis of MPS-induced anaphylaxis in this case, whereas BAT was not probably due to the prednisolone use before testing. This highlights the need to choose the appropriate procedure to diagnose glucocorticoid-induced anaphylaxis. The results in our patient suggest that BAT may be considered when IDST and SPT are negative. Further studies are necessary to clarify the best strategy for diagnosing glucocorticoid-induced anaphylaxis.

## Data Availability

The datasets analyzed during the study are available from the corresponding author by request.

## Abbreviations

BAT: Basophil activation test
BSP: Betamethasone sodium phosphate
DSP: Dexamethasone sodium phosphate
HCS: Hydrocortisone sodium succinate
IDST: Intradermal skin test
IgE: Immunoglobulin E
MPS: Methylprednisolone sodium succinate
mPSL: Methylprednisolone
PSS: Prednisolone sodium succinate
SPT: Skin prick test

## References

[1] Burgdorff T, Venemalm L, Vogt T, Landthaler M, Stolz W (2002). IgE-mediated anaphylactic reaction induced by succinate ester of methylprednisolone. Ann Allergy Asthma Immunol.

[2] Otani IM, Banerji A (2016). Immediate and delayed hypersensitivity reactions to corticosteroids: evaluation and management. Curr Allergy Asthma Rep.

[3] Steiner M, Harrer A, Himly M (2016). Basophil reactivity as biomarker in immediate drug hypersensitivity reactions-potential and limitations. Front Pharmacol.

[4] Aranda A, Mayorga C, Ariza A, Doña I, Blanca-Lopez N, Canto G (2010). IgE-mediated hypersensitivity reactions to methylprednisolone. Allergy.

[5] Ben Said B, Leray V, Nicolas JF, Rozieres A, Berard F (2010). Methylprednisolone-induced anaphylaxis: diagnosis by skin test and basophil activation test. Allergy.

[6] Nucera E, Lombardo C, Aruanno A, Colagiovanni A, Buonomo A, de Pasquale T (2011). ”Empty sella syndrome”: a case of a patient with sodium succinate hydrocortisone allergy. Eur J Endocrinol.

[7] Walker AI, Räwer HC, Sieber W, Przybilla B (2011). Immediate-type hypersensitivity to succinylated corticosteroids. Int Arch Allergy Immunol.

[8] Baeck M, Marot L, Nicolas JF, Pilette C, Tennstedt D, Goossens A (2009). Allergic hypersensitivity to topical and systemic corticosteroids: a review. Allergy.

[9] Younes M, Aquilina G, Castle L, Engel KH, Fowler P, Frutos Fernandez MJ (2020). Opinion on the re-evaluation of starch sodium octenyl succinate E1450 as a food additive in foods for infants below 16 weeks of age and the follow-up of its re-evaluation as a food additive for uses in foods for all population groups. EFSA J.

[10] Lehmann S, Ott H (2008). Glucocorticoid hypersensitivity as a rare but potentially fatal side effect of paediatric asthma treatment: a case report (1752–1947 (Print)). J Med Case Rep.

[11] Santos AF, Alpan O, Hoffmann HJ (2021). Basophil activation test: Mechanisms and considerations for use in clinical trials and clinical practice. Allergy.

[12] Sturm GJ, Kranzelbinder B, Sturm EM, Heinemann A, Groselj-Strele A, Aberer W (2009). The basophil activation test in the diagnosis of allergy: technical issues and critical factors. Allergy.

[13] Yamagata S, Tomita K, Sano H, Itoh Y, Fukai Y, Okimoto N (2012). Non-genomic inhibitory effect of glucocorticoids on activated peripheral blood basophils through suppression of lipid raft formation. Clin Exp Immunol.

[14] Yoshimura C, Miyamasu M, Nagase H, Iikura M, Yamaguchi M, Kawanami O (2001). Glucocorticoids induce basophil apoptosis. J Allergy Clin Immunol.

[15] Dykewicz MS, Lam JK (2020). Drug hypersensitivity reactions. Med Clin North Am.

